# Supplementary material for: The genetic basis of wing spots in Pieris canidia butterflies
Source: BMC Genomics. 2023 Apr 4;24:169. doi: 10.1186/s12864-023-09261-0 (PMC10074818; doi:10.1186/s12864-023-09261-0)
Supplement: Supplementary file 2 — Additional file 2: Table S1. List of differentially regulated genes common to both set of comparisons: i) spot vs cu1 and ii) spot vs proximal. Table S2. RNA concentrations obtained from dissected tissues for the 12 different libraries. Table S3. Summary of sequencing statistics and percentage of reads mapped to P. canidia genome using HISAT2. Figure S1. P. canidia genome size estimation using GenomeScope. Figure S2. Hierarchical clustering and heat map. Figure S3. Gene Ontology (GO) enrichment analyses for spot DEGs. Figure S4. Microdissections of early pupal wing tissues using dissecting tools fashioned from cut razor blades. [file 12864_2023_9261_MOESM2_ESM.docx]

**Supplementary Information**

**Table S1. List of differentially regulated genes common to both set of comparisons:** i) spot vs cu1 and ii) spot vs proximal. Up arrow symbol represents the upregulation of genes in wing tissues containing the ‘spot pattern’ while the down arrow symbol represents the downregulation of genes in wing tissues containing the ‘spot pattern’.

| Gene ID | Description | Direction of expression | |
| --- | --- | --- | --- |
|  |  | **‘spot vs cu1’** | **‘spot vs proximal’** |
| Pcan_09144 | *uncharacterized protein LOC111000960 isoform X1* | **↑** | **↑** |
| Pcan_09203 | *---NA---* | **↑** | **↑** |
| Pcan_09746 | *beta-mannosidase* | **↑** | **↑** |
| Pcan_11068 | *cytochrome P450 6k1-like* | **↑** | **↑** |
| Pcan_14498 | *uncharacterized protein LOC110997752* | **↑** | **↑** |
| Pcan_08069 | *unnamed protein product, partial* | **↓** | **↓** |
| Pcan_08073 | *tenascin-like isoform X3* | **↓** | **↓** |
| Pcan_08178 | *gloverin-like* | **↓** | **↓** |
| Pcan_09441 | *T-box transcription factor TBX6* | **↓** | **↓** |
| Pcan_09677 | *cecropin-like* | **↓** | **↓** |
| Pcan_12228 | *lysozyme-like* | **↓** | **↓** |
| Pcan_14073 | *serine protease inhibitor dipetalogastin-like isoform X2* | **↓** | **↓** |
| Pcan_05412 | *cuticle protein CP14.6-like* | **↑** | **↓** |
| Pcan_09799 | *larval cuticle protein LCP-17-like* | **↑** | **↓** |
| Pcan_09956 | *cAMP-dependent protein kinase catalytic subunit 1-like* | **↑** | **↓** |
| Pcan_13241 | *tiggy-winkle hedgehog protein* | **↓** | **↑** |

**Table S2. RNA concentrations obtained from dissected tissues for the 12 different libraries.**

| Sample Name | Treatment Group | Concentration (ng/µl) | Total Volume |
| --- | --- | --- | --- |
| S1 | Spot | 17.6 | 39 |
| S2 | Spot | 32.7 | 42 |
| S3 | Spot | 12.8 | 42 |
| S4 | Spot | 25.2 | 60 |
| P1 | Proximal | 13.1 | 42 |
| P2 | Proximal | 16.7 | 42 |
| P3 | Proximal | 21.6 | 42 |
| P4 | Proximal | 21.7 | 60 |
| C1 | Cu1 | 33.8 | 42 |
| C2 | Cu1 | 27.1 | 60 |
| C3 | Cu1 | 32.3 | 60 |
| C4 | Cu1 | 21.4 | 60 |

**Table S3. Summary of sequencing statistics and percentage of reads mapped to *P. canidia* genome using HISAT2.**

| Sample | Raw Reads | Effective Rate | Q20 (%) | Q30 (%) | GC Content (%) | Percentage of filtered reads mapping to genome (%) |
| --- | --- | --- | --- | --- | --- | --- |
| S1 | 54707539 | 97.15 | 98.06 | 94.19 | 50.04 | 96.40 |
| S2 | 62825665 | 97.21 | 98.21 | 94.53 | 51.38 | 92.71 |
| S3 | 48529341 | 97.25 | 98.16 | 94.39 | 51.48 | 96.98 |
| S4 | 95642456 | 98.27 | 98.32 | 94.91 | 47.30 | 96.91 |
| P1 | 51952490 | 97.88 | 98.25 | 94.63 | 50.39 | 96.70 |
| P2 | 50864060 | 98.80 | 97.97 | 93.94 | 49.97 | 96.20 |
| P3 | 48023578 | 97.89 | 98.20 | 94.47 | 50.59 | 96.88 |
| P4 | 94616878 | 98.48 | 98.03 | 94.29 | 47.46 | 96.64 |
| C1 | 61010193 | 97.83 | 98.09 | 94.28 | 51.40 | 96.85 |
| C2 | 86241796 | 98.76 | 97.92 | 94.08 | 48.01 | 96.72 |
| C3 | 98461684 | 98.52 | 98.07 | 94.42 | 49.04 | 96.74 |
| C4 | 98364038 | 98.21 | 98.47 | 95.29 | 48.12 | 96.82 |


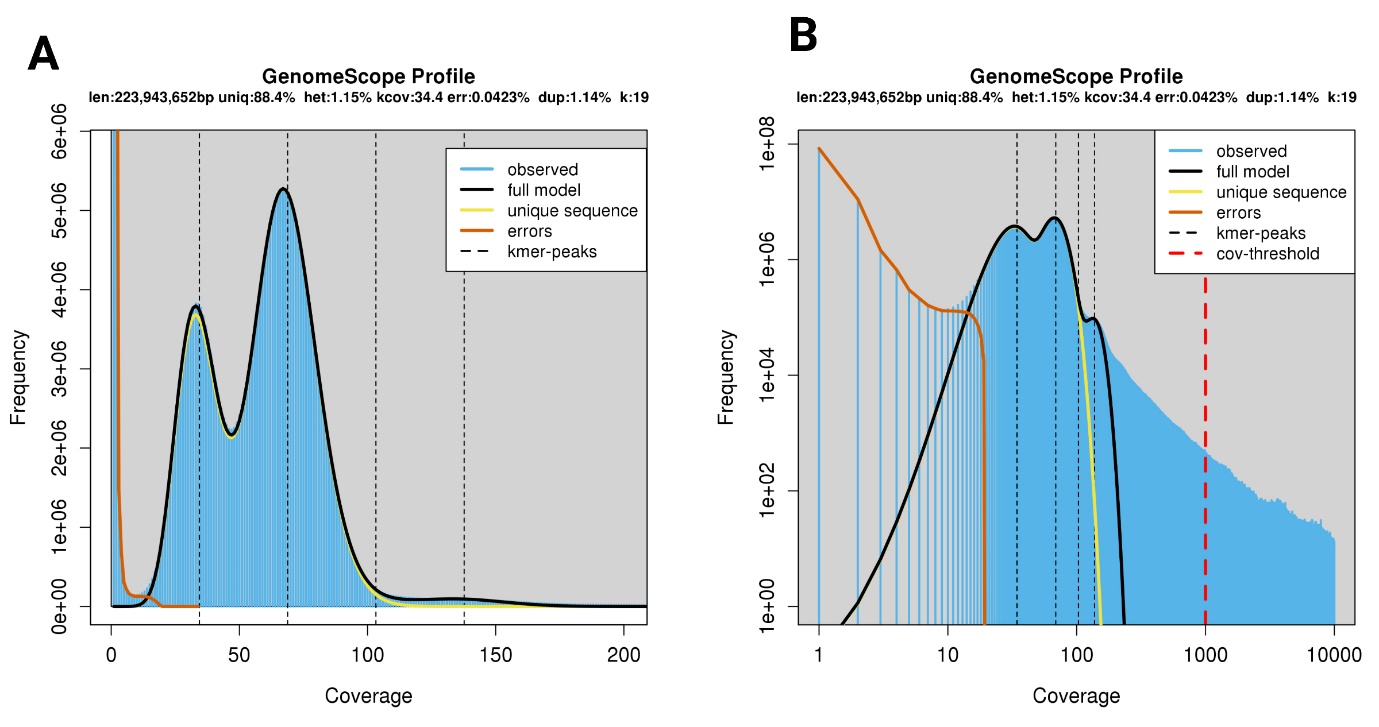


**Figure S1. *P. canidia* genome size estimation using GenomeScope.** We used Illumina short reads to estimate the genome size. We input the k-mer (k=19) analysis output from Jellyfish (Marçais & Kingsford, 2011) to estimate the haploid genome size. (A) Linear and (B) log plot of a k-mer spectral genome composition from *P. canidia* Illumina short read library. K-mer=19, Max K-mer coverage=1000. The estimated genome size was around 224 MB.


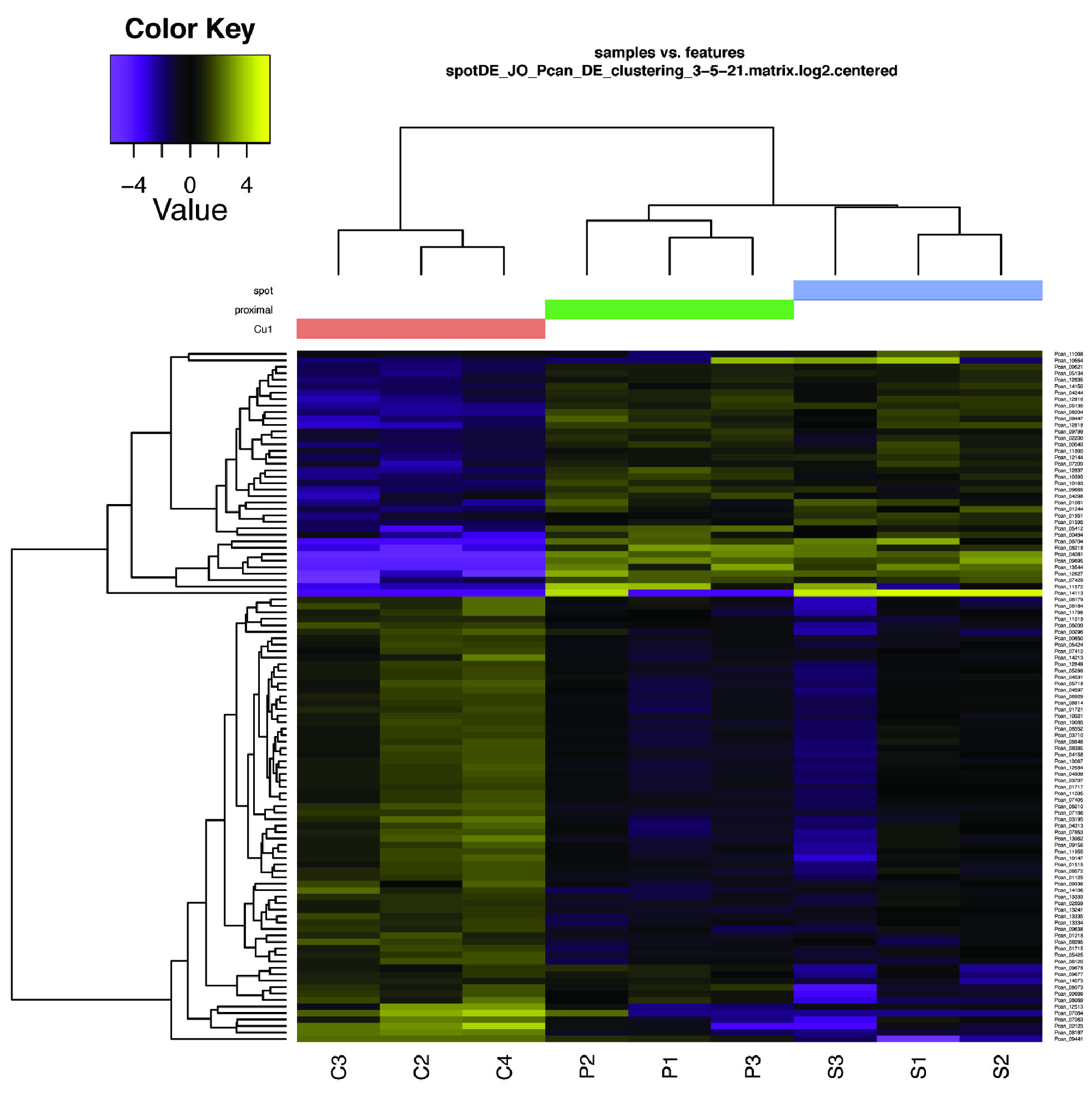
**Figure S2. Hierarchical clustering and heat map.** This clustering is based on the expression profiles of DE genes between “spot”, “proximal” and “cu1” groups for a set of significant genes (Padj <0.01).

*
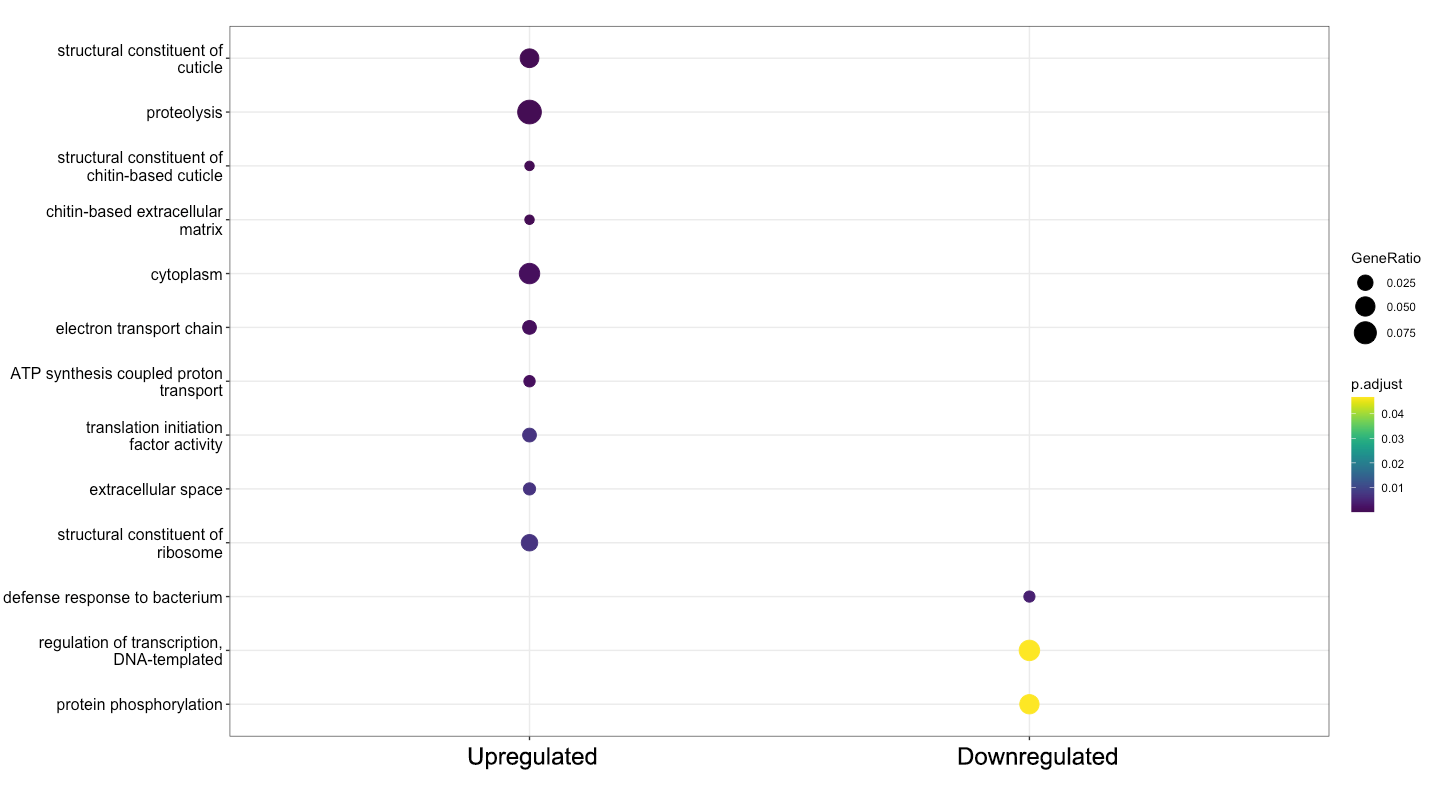
*

**Figure S3. Gene Ontology (GO) enrichment analyses for spot DEGs.** The function ‘*compareCluster’* within the Bioconductor package clusterProfiler 4.0 was used to determine over-represented GO terms in upregulated and downregulated DEGs, with the size of the dot denoting gene ratio and the colour of the dots representing the significance. P-values are adjusted by the Benjamini-Hochberg (BH) method.

**
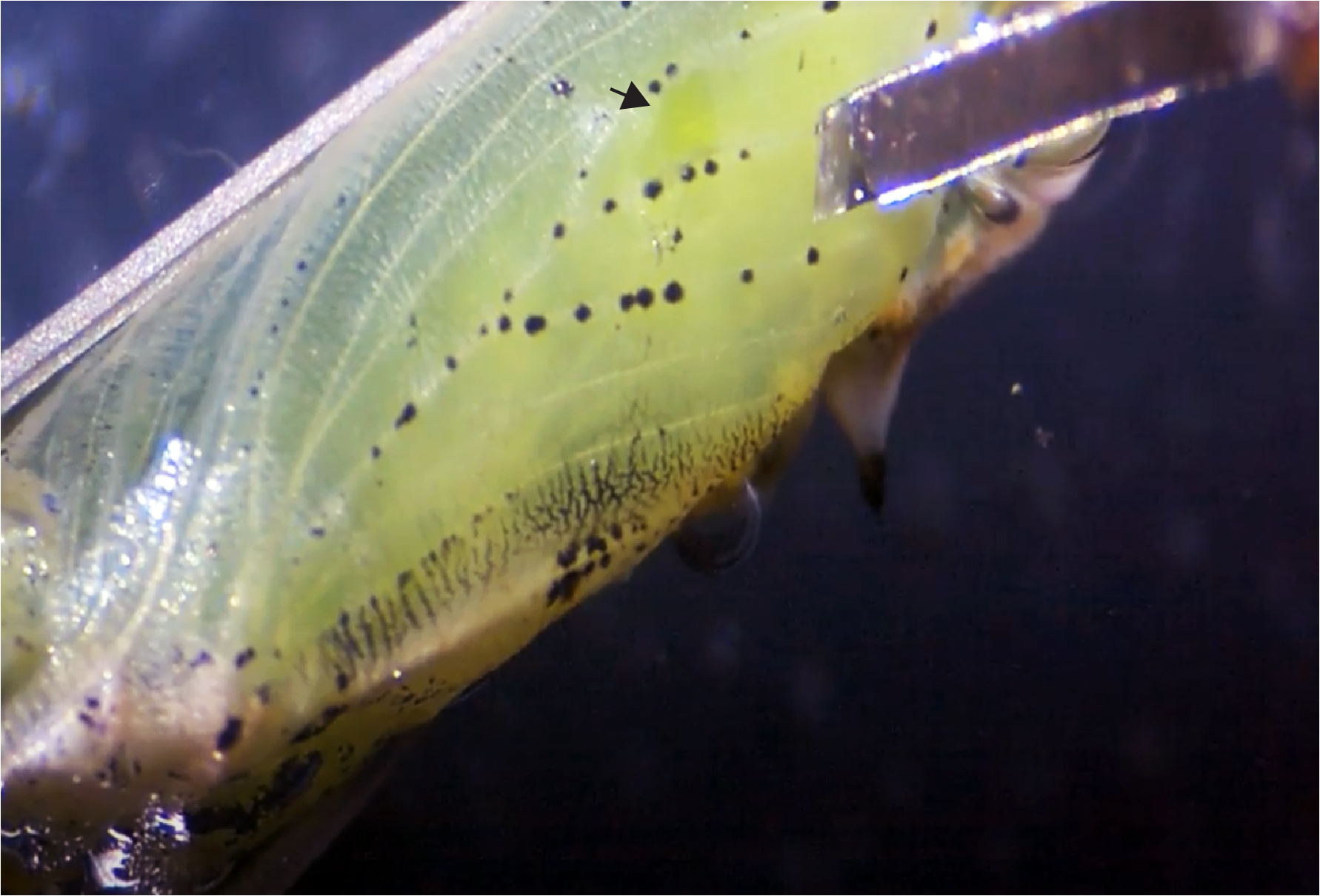
**

**Figure S4. Microdissections of early pupal wing tissues using dissecting tools fashioned from cut razor blades**. The black arrow denotes a piece of tissue that was already removed from the M3 wing sector.
